# Crop Enhancement of Cucumber Plants under Heat Stress by Shungite Carbon

**DOI:** 10.3390/ijms21144858

**Published:** 2020-07-09

**Authors:** Tae Yoon Kim, Hara Ku, Seung-Yop Lee

**Affiliations:** 1 Department of Biomedical Engineering, Sogang University, Baekbeom-ro 35, Mapo-gu, Seoul 04107, Korea; kimtaeyoon@sogang.ac.kr (T.Y.K.); haraku617@sogang.ac.kr (H.K.); 2 Department of Mechanical Engineering, Sogang University, Baekbeom-ro 35, Mapo-gu, Seoul 04107, Korea

**Keywords:** heat stress, cucumber, abiotic stress, natural carbon, shungite, antioxidant activity

## Abstract

Heat stress negatively impacts plant growth and yield. The effects of carbon materials on plants in response to abiotic stress and antioxidant activity are poorly understood. In this study, we propose a new method for improving heat tolerance in cucumber (*Cucumis sativus* L.) using a natural carbon material, shungite, which can be easily mixed into any soil. We analyzed the phenotype and physiological changes in cucumber plants maintained at 35 °C or 40 °C for 1 week. Our results show that shungite-treated cucumber plants had a healthier phenotype, exhibiting dark green leaves, compared to the plants in the control soil group. Furthermore, in the shungite-treated plants, the monodehydroascorbate content (a marker of oxidative damage) of the leaf was 34% lower than that in the control group. In addition, scavengers against reactive oxygen species, such as superoxide dismutase, catalase, and peroxidase were significantly upregulated. These results indicate that the successive pre-treatment of soil with a low-cost natural carbon material can improve the tolerance of cucumber plants to heat stress, as well as improve the corresponding antioxidant activity.

## 1. Introduction

The global climate has changed steadily over the past 100 years [1]. The geographical region of crop cultivation in South Korea has extended more than 90 km north in the same period. The temperature of the Earth has continuously risen and negatively affected the yield of essential crops necessary for human survival [2,3]. Meanwhile, the world population is growing exponentially and is estimated to be roughly 9 billion by 2050 [4]. In particular, all low- and mid-latitude countries are predicted to experience severe weather conditions in addition to global warming and high temperatures [5,6].

High temperatures result in a substantial decline in crop yields due to leaf wilting, physical damage to plant shoot and root growth, physiological disruption, biochemical changes, and reproduction problems [7,8]. It has been reported that an increase in the average temperature of just 1 °C results in a 4–10% decrease in crop yield [9]. Therefore, the development of new technologies is urgently needed to maintain crop yields under severe high-temperature stress.

To overcome high environmental temperatures, many solutions have recently been suggested by various countries. These include the building of smart farms that are controlled by computer systems based on artificial intelligence (AI) and the development of genetically engineered crops [10,11,12]. However, most of these technologies have numerous limitations, such as high cost, large space requirements, and human health and safety concerns [13]. Therefore, there is a need for the development of new economical technologies that meet the food demands of an ever-increasing global population, particularly under the challenges posed by global warming.

Recent studies have shown that a high content of inorganic carbon material in soil can have a positive or negative effect on plant growth [14,15,16]. In tomato (*Solanum lycopersicum* L.), the treatment of soil with single- or multi-carbon nanotubes (SWCNTs or MWCNTs) improved growth and resulted in multiple gene expression changes [17]. 

In this study, we tested a novel and cost-effective method for enhancing heat tolerance in plants using naturally derived shungite, which can be easily mixed into soil. Shungite, a natural inorganic carbon material, is excavated mainly in Russia and has been used as an eco-friendly construction material and water purification agent for over 100 years. It consists of 30–98% inorganic carbon, including 0.001–0.0001% fullerene structures [18]. Until recently, there have been few published studies on the effect of this carbon material on abiotic stress and antioxidant response. Interestingly, after a certain period of soaking carbon shungite stones in water, the water solution showed an increase in antioxidant levels via the reduction of intracellular reactive oxygen species (ROS) production and the enhancement of antioxidant enzyme activities, including glutathione peroxidase (GPx), superoxide dismutase (SOD), and myeloperoxidase (MPO), in animal cells [19]. This points to an important clue to the antioxidant responses of other living organisms, because numerous studies have found a significant number of homologs at the animal and plant genetic level that frequently exhibit similar functions [20].

We measured the activity of scavenger-related ROS produced by heat stress. The main types of ROS are superoxide (O_2_), hydroxyl (OH^-^) ions, hydrogen peroxide (H_2_O_2_), and singlet oxygen [21,22,23]. Under normal conditions, most intracellular free radicals are quickly degraded or stabilized by numerous antioxidants [24]. During stress, ROS levels increase exponentially and exceed the ability of removal by scavengers. The residual ROS damages DNA, proteins, and lipids, which are major components of cell life [25,26]. Plant scavengers are classified into enzymatic and non-enzymatic antioxidants depending on their functional properties. Among the plant scavengers, superoxide dismutase (SOD), catalase (CAT), and peroxidase (POD) are involved in heat stress responses [27].

Heat shock proteins (HSPs) are an important family in the plant life cycle, as they protect plants from biotic and abiotic stresses. These stress-responsive biomolecules act as molecular chaperones [28]. While HSPs were first characterized because of their response to high temperatures, they have since been found to play roles in normal, non-stressed cells. They are produced at specific stages of the cell cycle or are involved in normal plant growth and development, like flower, seed, and fruit development, as well as tuberization and nutrient uptake [29,30,31,32,33,34]. HSPs are found in various cell compartments, such as the cytoplasm, nucleus mitochondria, chloroplasts, and endoplasmic reticulum [9]. HSPs are grouped into different classes based on their molecular weight in kilodaltons (kDa) [35,36,37,38]. Some studies have reported that HSP gene expression positively regulates the activity of protective enzymes. In *Arabidopsis thaliana*, overexpression of the *AtHSP17.8* gene can enhance SOD enzymatic activity and, in tobacco, *NtHSP16.9* can increase the activities of POD, CAT, and SOD [39]. In cucumber (*Cucumis sativus L*.), *CsHSP45.9* and *CsHSP70* are known to be responsible for tolerance to heat stress [40]. Most studies on the activities of antioxidant enzymes have yielded inconsistent results because of differences in plant species, genotype, stress level, stress duration, and plant developmental stage [26,41].

The cucumber is an economically important plant under protected cultivation and is sensitive to heat stress. In this study, we investigated the effect of shungite on cucumber cultivation, and identified differentially expressed proteins influenced by heat stress to observe the heat tolerance of cucumber plants and the corresponding antioxidant activity.

## 2. Results

### 2.1. Enhanced Heat Tolerance of Shungite-Treated Plants

Plant growth occurs in three stages, namely cell division, differentiation, and elongation. In prolonged conditions of high temperature stress, the photosynthetic efficiency decreases, resulting in growth inhibition. Prolonged high temperature results in poor leaf expansion, stem growth, flowering, and yield.

**Figure 1:**
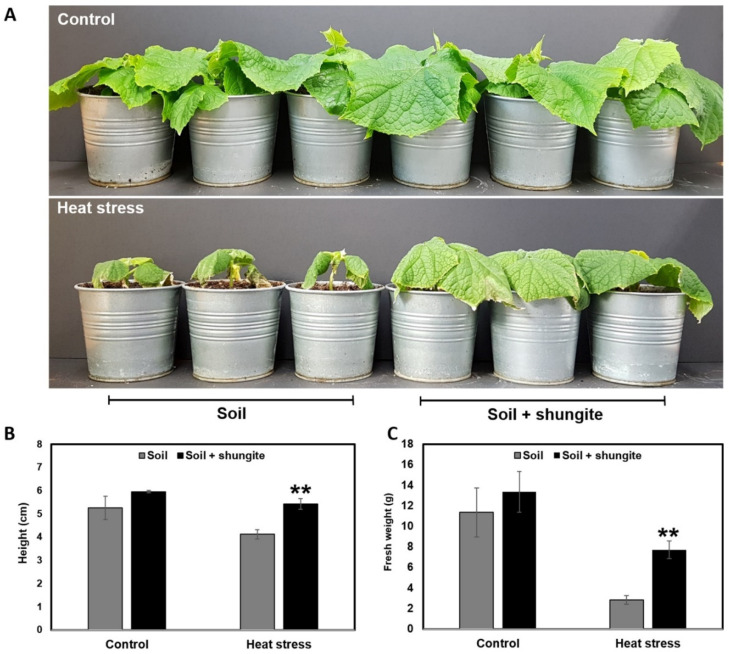
Effects of shungite on heat tolerance in cucumber. (**A**): Less drooping in leaves of plants exposed to shungite. Representative plants that underwent the two-step heat treatment; (**B**): Comparison of plant heights; (**C**): Comparison of fresh weight. Error bars represent mean ± standard deviation (SD), (*n* = 10). Asterisks indicate statistical significance (Student’s *t*-test, ** *p* ≤ 0.05).

Our previous study showed that shungite has a positive effect on cucumber plants under drought stress [5]. In the present study, we tested the effect of shungite on heat stress in cucumber plants. For normal growth conditions, plants were treated with tap water at two-day intervals for a total of 12 days. Following this, two steps of heat treatment were applied to plants at the four-leaf stage. After two weeks, stress symptoms, such as drooping in xanthic leaf plants, were observed in only the control, normal soil group. In general, plant drooping occurs with curling edges and yellow discoloration under water deficit and heat. This is a defense mechanism because wilted leaves help the plant to reduce surface area and, subsequently, water loss to the atmosphere. In contrast, cucumbers grown in shungite-containing soil displayed darker green-colored leaves and a healthier phenotype than the control group (Figure 1A). For a more accurate comparison, eight plants were randomly selected from each group and compared for height and fresh biomass. The control group displayed about 30% smaller height and 70% lower fresh weight than the shungite additive group grown in high temperatures (Figure 1B,C). These results indicate that shungite may enhance plant tolerance against heat stress.

### 2.2. Effects of Shungite on Heat Tolerance

Unexpected and sustained heat stress rapidly leads to the accumulation of ROS in plant cells. If ROS levels exceed the threshold that plants can respond to, they cause oxidative damage, such as cell membrane degradation. malonyldialdehyde (MDA) and electrical conductivity (EC) levels change rapidly and are therefore well-known markers of oxidative damage for various cell types, including in plants [42].

**Figure 2:**
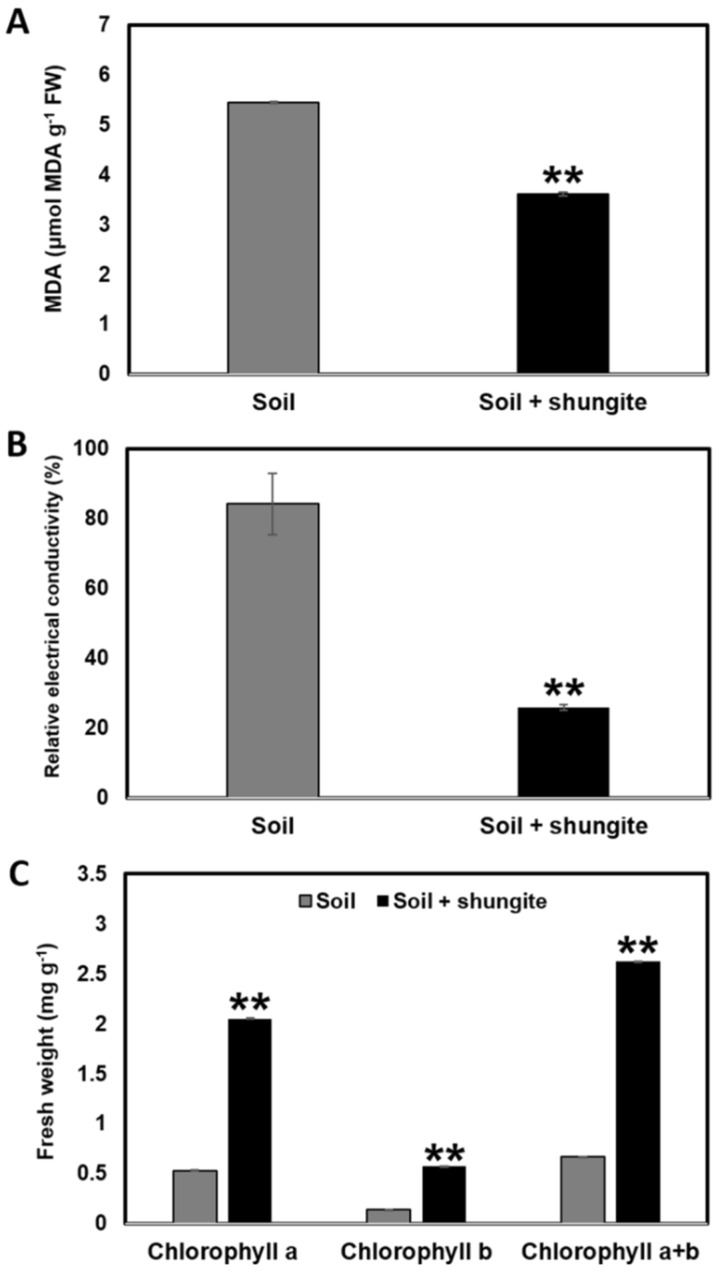
Effects of shungite on physiological indicators of heat tolerance in cucumber. (**A**): Comparative analysis of malonyldialdehyde (MDA) accumulation in the leaves of cucumber; (**B**): Relative electrical conductivity levels in cucumber leaves; (**C**): The total content of chlorophyll in the same leaf region (3 cm^2^). All experiments were performed in triplicate, and the averages are graphed. Error bars represent the mean ± SD. Asterisks indicate statistical significance (Student’s *t*-test, ** *p* ≤ 0.05).

In order to evaluate the effect of shungite on cucumber plants exposed to heat stress, the MDA, EC, and chlorophyll contents in leaves were determined. The MDA concentration of the plants in the shungite-containing group was about 3.3 nmol/g per fresh weight, which was about 40% lower than the levels in the control plants (Figure 2A). The EC value in the shungite treatment group was 22%, which was 73% lower than the value in the control group (Figure 2B). In addition, the amount of total chlorophyll in a given 3 cm^2^ area was fivefold higher than that in the control group (Figure 2C). These results could be due to reduced oxidative stress.

### 2.3. Gene Expression Changes and Antioxidant Activity

**Figure 3:**
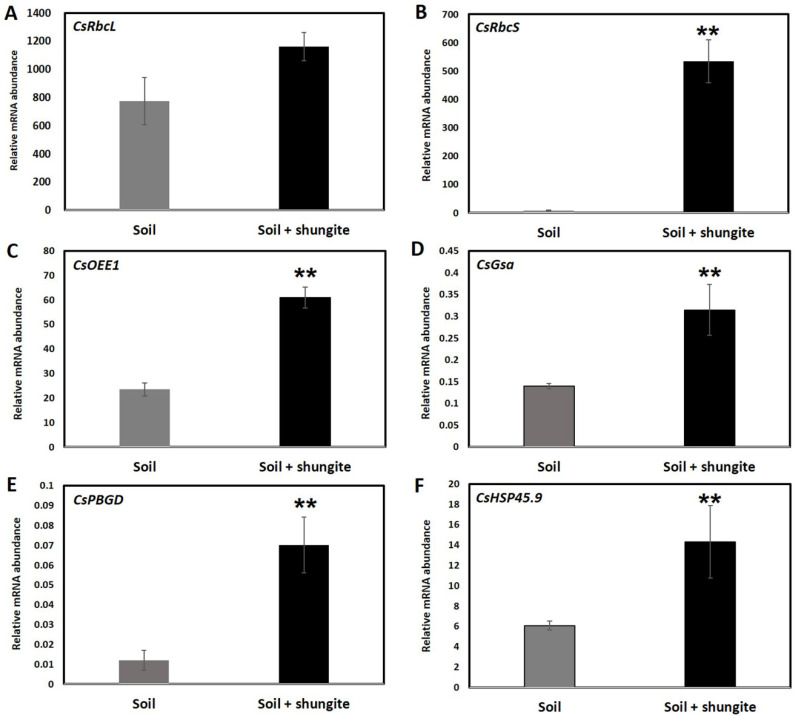
Effects of shungite and/or heat stress treatment on *CsRbcL*, *CsRbcS*, *CsOEE1*, *CsGsa*, *CsPBGD*, and *CsHSP45.9* transcripts in leaves of cucumber were determined using real-time PCR (**A**–**F**). The *γ*-axis represents relative expression levels normalized by the elongation factor 1-alpha (*CsEF1α*) gene (*Csa006172*). Three independent experiments with three biological replicates were averaged (± SD). Statistically significant differences between control and shungite-treated groups at corresponding time points are indicated by asterisks (Student’s *t*-test, ** *p* ≤ 0.05).

As both temperature and shungite induce significant changes in membrane peroxidation (Figure 1 and Figure 2), we analyzed the transcripts of genes involved in cellular defense and stress response. Many photosynthesis and photosynthetic pigment metabolism progress-related proteins were altered by shungite treatment. To verify these results, we analyzed the expression pattern of three photosynthesis-related genes (rubisco large subunit (*CsRbcL*), rubisco small subunit (*CsRbcS*), and oxygen-evolving enhancer protein 1 (*CsOEE1*)), two photosynthetic pigment metabolism-related genes (glutamate-1-semialdehyde 2,1-aminomuase (*CsGsa*) and porphobilinogen deaminase (*CsPBGD*)) [43] and one gene involved in protein protection (heat shock protein 45.9 (CsHSP45.9)), shown in Figure 3A–F. The transcript levels of these genes were largely influenced by the shungite and temperature treatments. In the shungite-treated cucumber plants, *CsRbcL*, *CsRbcS*, *CsOEE1*, *CsGsa*, *CsPBGD*, and *CsHSP45.9* transcripts were dramatically upregulated by 1.5-, 55.0-, 2.3-, 7.0-, 2.9-, and 2.3-fold, respectively (Figure 3). The primers used for real-time PCR are listed in Supplementary Table S1.

**Figure 4:**
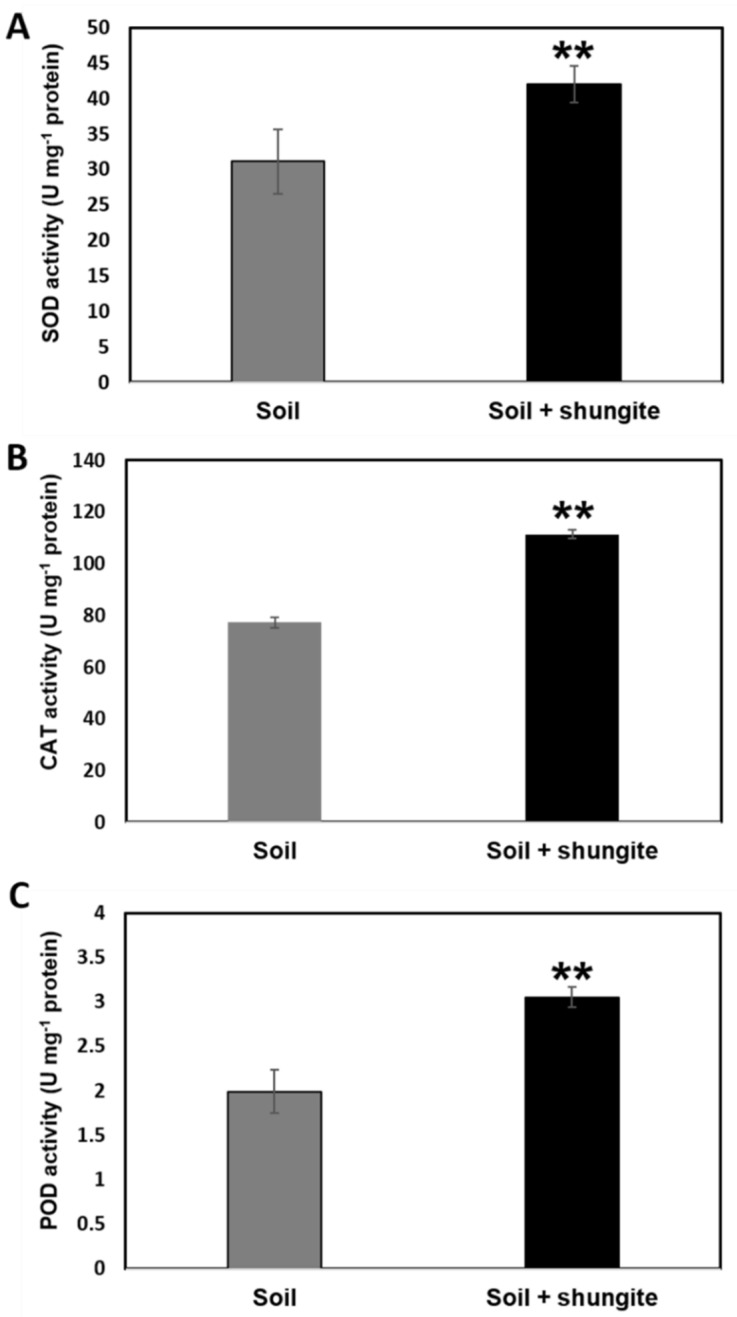
Comparison of reactive oxygen species scavenger activity under heat stress. The (**A**): Superoxide dismutase (SOD), (**B**): Catalase (CAT), and (**C**): Peroxidase (POD) activities in cucumber plant leaves. Experiments were performed using three randomly collected leaves from eight plants on day 14, at which point severely wilted plants were observed in the control group. All experiments were performed in triplicate, and the averages are graphed. Error bars represent the mean ± SD. Asterisks indicate statistical significance (Student’s *t*-test, ** *p* ≤ 0.05).

ROS production in plants is highly responsive to stress conditions [44]. Tolerance to heat-induced oxidative stress in crop plants has been associated with an increase in antioxidative capacity [45]. Among the numerous enzymatic and non-enzymatic scavengers, SOD, CAT, and POD reduce the levels of heat stress-induced ROS [9,34,37]. We investigated the effect of shungite on the enzymatic scavenger system in response to heat stress by determining the activity of SOD, CAT, and POD in randomly collected leaves. As shown in Figure 4A, shungite could enhance SOD activity in leaves, peaking at 41 U/mg compared with only 30 U/mg in the control. Moreover, in shungite-treated cucumber plants, CAT and POD activity in leaves was 1.5- and 1.6-fold higher than that in the control plants (Figure 4B,C). Taken together, these results indicate that the shungite-treated group was less affected by heat stress than the control group.

### 2.4. Modulation of the Antioxidant Defense System Associated with Heat Stress

**Figure 5:**
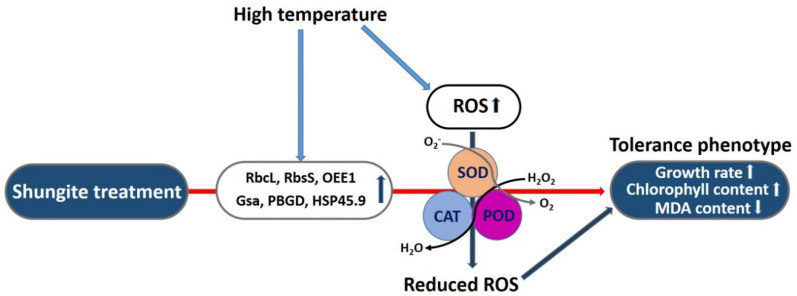
Schematic diagram of shungite-mediated tolerance under heat stress in cucumber. Red solid arrows show plants directly influenced by shungite. Heat stress causes the development of ROS and various types of cell and organ damage, including the degradation of membrane, DNA, and protein. The addition of shungite to soil activates genes related to photosynthesis and the defense response, increases ROS scavenger activity, and reduces cellular or organ damage. This leads to higher growth rates and chlorophyll content.

ROS usually arise in living organisms as a result of aerobic metabolism. However, metabolic imbalances caused by environmental changes, including heat stress, accelerate the accumulation of ROS [46,47]. Interestingly, although ROS, such as H_2_O_2_ or O_2_, are considered essential signal transduction molecules, they are also toxic and able to cause extensive cellular damage and photosynthesis inhibition [48,49]. Therefore, we determined the relative expression level of rubisco large subunit (*CsRbcL*), rubisco small subunit (*CsRbcS*), oxygen-evolving enhancer protein 1 (*CsOEE1*), glutamate-1-semialdehyde 2,1-aminomuase (*CsGsa*), and porphobilinogen deaminase (*CsPBGD*). These genes are related to photosynthesis or photosynthetic pigment metabolism. We found that the expression levels increased dramatically in the plants exposed to natural shungite (Figure 5). Furthermore, the accumulation of high levels of heat stress response transcripts, such as *HSP45.9*, in cucumber plants reflects the importance of ROS in acclimation pathways during combined stresses [50]. ROS-related damage through ROS accumulation is reduced by the antioxidant system, including ROS scavengers and expanded levels of antioxidants, which is referred to as the Halliwell–Asada cycle [51]. SOD, which constitutes the first level of defense against superoxide radicals, is one of the main enzymes of the antioxidant defense system. SOD catalyzes the reaction of O_2_ and H_2_O_2_, which are removed by POD and CAT [51]. Several studies have shown that the ability of plants to control ROS production and removal is related to higher tolerance to various abiotic stresses [52]. For this reason, shungite may enhance the heat tolerance of plants by modulating the enzymatic activity of ROS scavengers such as SOD, CAT, and POD (Figure 5).

## 3. Discussion

Recently, several pieces of research have noted that a high content of carbonaceous materials and MWCNTs have a positive impact on plant growth [15,16,17]. Shungite is a natural element containing a high composition of carbon, including fullerene. To verify the effectiveness of shungite on heat stress in cucumber plants, morphological, physiological, and biochemical analyses have been conducted in this study. After applying a two-step heat treatment for 14 days, the shungite treatment group showed a more tolerant phenotype compared to the control soil group. The adult leaves of cucumber grown in normal soil became yellow and dry, which is a sign of chlorophyll degradation; however, shungite-treated cucumber plants maintained a healthier phenotype with green leaves (Figure 1). These results indicate that shungite may improve heat tolerance in plants as a simple soil additive.

Under a heat stress environment, plants not only showed growth inhibition, but in severe cases, the chloroplasts were destroyed, leading to plant death. Furthermore, plants underwent dehydration to reduce their biomass [53,54]. One of the main causes of this phenomenon is the creation of ROS. ROS are mainly produced in the mitochondria, chloroplasts, and peroxisomes as the by-products of various metabolic pathways. ROS are short-lived, but as strong oxidants, they can have a fatal effect on the regulation of the physiological activity of intracellular DNA and protein. Therefore, their effect on growth was assessed using visual observation, fresh weight, chlorophyll content, and MDA measurement. MDA accumulation is widely used as a marker of oxidative stress in animals and plants [55,56]. We found that the MDA levels of the plants in the shungite group were 37% lower than that in the control group (Figure 2A). Further assays were employed to compare between the groups. The extent of leaf cell damage was assessed by measuring the EC levels and total chlorophyll content. We found that the shungite-treated plants had a less affected phenotype when exposed to heat stress (Figure 2B,C).

A major physiological response related to high temperature is photosynthetic efficiency. Thus, the enzymes for energy distribution and carbon metabolism, in particular rubisco, are significantly affected by heat stress. Heat-tolerant crop species have higher photosynthetic efficiency when exposed to high temperatures. Therefore, heat stress disturbs the expression levels of plant genes, especially those coding proteins related to photosynthesis [43]. For this reason, we selected and analyzed the accumulation rate of three photosynthesis-related genes (*CsRbcS*, *CsRbcL*, and *CsOEE1*) and two photosynthetic pigment metabolism-related genes (*CsGsa* and *CsPBGD*) [9,36,40,43]. Our results show that the expression levels increased from 1.5- to 500-fold in the shungite treatment group under heat treatment conditions compared to the control. In general, changes in gene expression vary widely depending on plant species. Even within the same species, gene expression levels are different depending on the sampling factors and stress conditions. Therefore, the results in Figure 3 only present the relative changes in the stress-related genes for the Korean cucumber (cv. *Eun Sung*) at the given experimental conditions. In the case of the heat shock protein *CsHSP45.9*, it is known to control the regulation of ROS scavengers under heat stress via the heat stress factor (HSF)–HSP–heat shock factor binding protein 1 (HSBP1) pathway. The HSFs transfer the ROS signal to downstream transcription factors through the mitogen-activated protein kinase (MAPK) signaling pathway [57]. The MAPK signaling pathway activates downstream redox-sensitive transcription factors. In response to oxidative stress, these transcription factors are coordinated through specific oxidative stress-sensitive cis-elements in gene promoters, which primarily encode antioxidant enzymes and non-enzymatic antioxidants [58]. Multitudinous antioxidant enzymes (SOD, CAT, and POD) primarily act as the scavengers of ROS under heat stress in plants [59,60,61,62]. The activities of different antioxidant enzymes are temperature sensitive and activation occurs at different temperature ranges, but the activities of these enzymes increase with temperature [63]. Previous studies observed that SOD and CAT showed an initial increase before declining at 50 °C [63,64]. We found that the activity of SOD, CAT, and POD in the shungite-treated soil group was 20–34% higher than in the cucumber plants in the control group. In general, our investigations support that antioxidant activity is related to increased SOD, CAT, and POD, and allowed cucumber plants to reduce stress-induced oxidative damage.

In general, most plants have the ability to absorb water and minerals from stones [65,66]. The water-soluble mineral components absorbed by plants, in turn, benefit natural consumers, including humans. Natural shungite is composed mainly of carbon (28–99%). Interestingly, after incubating shungite and fullerene stones in water, shungite water solutions were reported to have an antioxidant effect via the reduction of intracellular ROS production and the enhancement of antioxidant enzyme activities in animal cells [19]. The study also reported substantial improvements in the skin parameters (moisture, elasticity, roughness, pore size, pigmentation, and wrinkles) of shungite-treated groups, which were related to the recovery of total white blood cells. Consistently, ROS-scavenging enzymes, such as GPx and SOD, in the shungite treatment group showed increased activity levels of 25% and 33% in skin lysate, respectively. Synthetically, these results led to an antioxidant and anti-inflammatory effect against UVB-induced skin damage in hairless mice [19].

## 4. Materials and Methods

### 4.1. Cucumber Plant Culture

Peeled cucumber seeds of “Eun Sung” (Farm Hannong, Seoul, South Korea) were surface-sterilized in 70% ethanol for 1 min, and then in 2% hypochlorite solution for 15 min. After three washings, the seeds were planted in basal Murashige and Skoog (MS) medium (Duchefa Biochemie, RV Haarlem, Netherlands) for 3 d, containing 3% sucrose, 500 mg/L 2–(N–morpholino) ethanesulfonic acid (MES), and 0.8% phyto agar, at pH 5.8 and 23 ± 1 °C in the dark. The plates were moved to a condition of 16 h-long daylight and allowed to expand for another 4 d. Total seedlings were shifted to normal soil and were grown to the four-leaf stage (Cocopeat 68%, peat moss 14.73%, pearlite 7%, vermiculite 6%, zeolite 4%, Seoul Bio, Chungcheongbuk-do, South Korea).

### 4.2. Evaluation of Shungite-Treated Plants Under Heat Stress

Natural shungite has 28–30% carbon content, 2.3–2.4 g/cm^3^ specific gravity, 0.5% porosity, 1000–1500 kg f/cm^2^ compressed strength, and 1100–1200 kg/m^3^ density [19]. Prepared cucumber seedlings were transplanted with normal soil or soil mixed with shungite powder (0.067% carbon content, 2 g(v)/L(v), 300 mesh, outer diameter < 2 mm, Karelia, Russia) into iron pots (upper diameter 10.5 cm; height 9.5 cm). A carbon content of 0.067% showed the strongest phenotype in our previous plant growth and drought tolerance test [5]. Thirty seedlings per group were irrigated at two-day intervals with 80 mL of tap water for a total of 28 days to maintain an optimal level of humidity. For the two-step heat stress treatment, the first step consisted of a week at 35 ± 1 °C during the day and 30 ± 1 °C during the night. The second step consisted of one week at 40 ± 1 °C during the day and 35 ± 1 °C during the night. In all growth conditions, 30–40% humidity and 600 μmol photons·m^−2^·s^−1^ of light were maintained for 16 h during the day. The heights and fresh weight were determined by a random collection of ten individuals from each group. The lowest and highest measurements were excluded and the readings of the remaining eight plants were averaged. 

### 4.3. Measurement of Malonyldialdehyde Content, Relative Electrical Conductivity, and Chlorophyll Content

The MDA content was determined in leaves 14 d after the two-step heat stress treatment [67]. Three leaves were randomly selected from each group of ten plants; a 0.3 g fresh weight (FW) sample was homogenized in 5 mL of 5% trichloroacetic acid (TCA). Next, the homogenate was centrifuged for 15 min at 8000× *g*. The supernatant from each sample was combined with 2.5 mL of thiobarbituric acid (TBA), and the mixture was heated for 20 min in a 100 °C water bath, and then cooled immediately on ice. The mixture was then centrifuged for 5 min at 10,000× *g* and the absorbance of the corresponding supernatant was measured at 532 and 600 nm. Based on its molar extinction coefficient (155 mM^−1^·cm^−1^), the concentration of MDA in cucumber leaves was obtained and denoted as μmol MDA·g^−1^·FW.

The relative EC percentage in cucumber leaves was measured according to the process defined by Yang et al. [68]. Three leaves were gathered for each test, then minced per 0.1 g fresh weight (FW) sample, put in a 50 mL Falcon tube, and combined with 10 mL of distilled water. The initial EC (S1) was calculated after the mixture was incubated in a calorstat set at 32 °C for 2 h. Following this, the mixture was boiled for 30 min at 100 °C and then cooled to room temperature to determine its final EC (S2). Distilled water with an electrical conductance value of zero (S0) was used as the background value. The relative electrical conductivity (REC) was determined using Equation (1):

REC = (S1 − S0)/(S2 − S0) × 100 (1)

The chlorophyll content of leaves was estimated 14 d after the two-step heat stress treatment using a previously described method [69]. In each group, three leaves were collected randomly from three separate plants. Samples of 0.3 g fresh weight were harvested at 4 °C, cut into segments of 3 cm × 3 cm, and incubated with 100% ethanol overnight. The mixture was then centrifuged for 5 min at 13,000 rpm, and the supernatant was collected and measured using a spectrophotometer at 645 and 663 nm. The concentration of chlorophyll was calculated using the Arnon equations [70], as follows in Equations (2)–(4):

Chlorophyll a (µg/mL) = 12.7 × A663 − 2.69 × A645 (2)

Chlorophyll b (µg/mL) = 22.9 × A645 − 4.68 × A663 (3)

Total chlorophyll (µg/mL) = 20.2 × A645 + 8.02 × A663 (4)

### 4.4. RNA Extraction and Real-Time PCR Analysis

The cucumber leaves were immediately frozen in liquid nitrogen after harvesting and stored at −80 °C prior to RNA extraction. Total RNA from the cucumber leaves was extracted using the RNeasy Kit (QIAGEN, Hilden, Germany) according to the manufacturer’s instructions. DNA contamination was eliminated with a purifying column and DNAse. Using a QuantiTech SYBR Green RT-PCR kit (QIAGEN), 1 mg of total RNA was used for reverse transcription and the quantitative real-time PCR (qRT-PCR) assay was performed. Accurate measurement data were obtained using the LightCycler96 system (Roche Life Science, Penzberg, Germany). The reaction conditions were as follows: denaturation at 95 °C for 10 min, followed by 40 cycles of denaturation at 95 °C for 20 s, annealing at 58 °C for 20 s, and extension at 72 °C for 30 s. The transcript levels of *CsRbcL*, *CsRbcS*, *CsGsa*, *CsPBGD*, *CsOEE1*, and *CsHSP45.9* were normalized to *CsEF1α (Csa006172)*, and mRNA was quantified using the LightCycler96 software (version 1.1.0.1320). Experiments were performed using three separate biological samples and three experimental replications for each qRT-PCR reaction. The primer sequences were modified based on a previous study [43]. The primer sequences are provided in the supporting information section (Table S1).

### 4.5. Determination of Antioxidant Activity

The activity of the three antioxidant enzymes (SOD, CAT, and POD) in cucumber tissues was assessed using commercial assay kits. Three leaves were collected randomly from three of the ten plants; after harvesting, samples were frozen at −20 °C and ground in the presence of ice. Then, 1 mL of 50 mM ice-cold phosphate buffer (pH 7.8) containing 1 mM ethylene diamine tetra acetic acid (EDTA) was applied to each ground sample. The samples were vortexed for 5 min and incubated for 1 h in ice. The homogenate was then centrifuged for 15 min at 15,000× *g* and 4 °C. The obtained supernatant was used to perform enzyme assays.

The SOD Assay Kit-WST (Dojindo, Kumamoto, Japan) was used to test the SOD activity in cucumber samples. Mono sodium salt (2-(4-iodophenyl)-3-(4-nitrophenyl)-5-(2,4-disulphophenyl)-2H-tetrazolium; WST) is reduced by the superoxide anion, creating yellow formazan that can be measured using a spectrophotometer at 450 nm. The presence of antioxidants prevents the development of yellow WST formation. Briefly, cucumber extract was mixed with the WST solution, and then samples were treated with the enzyme solution and incubated at 37 °C for 20 min. The absorbance was measured with a spectrophotometer at 450 nm. The inhibition rate of formazan formation was calculated as in Equation (5):

((A_blank 1_ − A_blank 2_) − (A_sample_ − A_blank 2_))/(A_blank 1_ − A_blank 3_) × 100 (5)

The inhibition rates were changed to SOD enzymatic levels at a specific protein. All tests were replicated three times.

The CAT enzymatic activity was calculated using the CAT Assay Kit (Biomax, Korea). Catalase reduces H_2_O_2_ into water and oxygen. Red resorufin is formed when H_2_O_2_ reacts with the reagent probe and horseradish peroxidase. The amount of reduced resorufin within the sample correlates to the amount of antioxidant activity. The absorbance of resorufin was measured using a spectrophotometer at 560 nm. All experiments were replicated three times.

The POD enzymatic activity in each sample was calculated using the POD Assay Kit (Biomax, Korea). The POD values were determined using a probe and horseradish peroxidase. The reduced resorufin of the final reaction product was calculated using a spectrophotometer at 560 nm.

### 4.6. Statistical Analysis

All experiments were performed in a randomized design and conducted in triplicate, and the means were graphed. All data were analyzed using Minitab software (version 18, State College, PA, USA) and the difference between means was determined using the Student’s *t*-test at *p* ≤ 0.05. 

## 5. Conclusions

Based on results of plant growth and heat tolerance in cucumber (*Cucumis sativus* L.), this study confirmed that the low-cost natural carbon material shungite has strong and direct antioxidant activity via the enhancement of SOD, CAT, and POD scavengers. Shungite is also effective in increasing heat tolerance in plants for crop enhancement, which is associated with a higher chlorophyll content, leaf area, and rate of photosynthesis. This enhanced phenomenon is attributed to a high accumulation of heat response genes, such as *HSP45.9*, as well as the upregulation of photosynthetic and photosynthetic pigment metabolism-related genes. These findings may provide an innovative solution to major agricultural challenges caused by global warming.

## Supplementary Materials

The following are available online at https://www.mdpi.com/1422-0067/21/14/4858/s1, Table S1: Primers used for real time RT-PCR assays.
