# Supplementary material for: Crop Enhancement of Cucumber Plants under Heat Stress by Shungite Carbon
Source: Int J Mol Sci. 2020 Jul 9;21(14):4858. doi: 10.3390/ijms21144858 (PMC7402313; doi:10.3390/ijms21144858)
Supplement: Supplementary file 1 [file ijms-21-04858-s001.pdf]

Supplementary Materials

Table S1. Primers used for real time RT-PCR assays.

Gene	Primer Pairs
CsEF1α	F : 5'-GATGATTTGCTGCTGCAACAAGATG-3'<br>R : 5'-TTGTACCAGTCAAGGTTGGTCGACC-3'
CsRbcL	F : 5′-AGCCTGTTGCTGGAGAAG-3′<br>R : 5'-AGGGCGACCATACTTGTT-3'
CsRbcS	F : 5'-GCCTCAAATCTTCCGCTGGT-3'<br>R : 5'-AATCCGCTTCCGATGTCGAAT-3'
CsOEE1	F : 5'-GCGGCTACCCTTATGCAAC-3'<br>R : 5'-ATCTTAGCAGCATCGGCAAAC-3'
CsGsa	F : 5'-CGGCTTGCTCGTGCTT-3'<br>R : 5'-CTGGTTCTTTCAATCGCTTTA-3'
CsPBGD	F : 5'-TCATGGCGTTGGTCTCATAAG-3'<br>R : 5'-ACAGGCAATCCCAATAGCTCC-3'
CsHSP45.9	F : 5'-TCCTGGCTGTCAAGGTTCTGG-3'<br>R : 5'-CGACGAGAGACAAGGTATGCT-3'

F indicates forward and R indicates reverse.
